# Impact of high ambient temperature on unintentional injuries in high-income countries: a narrative systematic literature review

**DOI:** 10.1136/bmjopen-2015-010399

**Published:** 2016-02-11

**Authors:** Eveline Otte im Kampe, Sari Kovats, Shakoor Hajat

**Affiliations:** Department of Social and Environmental Health Research, London School of Hygiene and Tropical Medicine, London, UK

**Keywords:** injuries, hot weather, high income countries, review

## Abstract

**Objectives:**

Given the likelihood of increased hot weather due to climate change, it is crucial to have prevention measures in place to reduce the health burden of high temperatures and heat waves. The aim of this review is to summarise and evaluate the evidence on the effects of summertime weather on unintentional injuries in high-income countries.

**Design:**

3 databases (Global Public Health, EMBASE and MEDLINE) were searched by using related keywords and their truncations in the title and abstract, and reference lists of key studies were scanned. Studies reporting heatstroke and intentional injuries were excluded.

**Results:**

13 studies met our inclusion criteria. 11 out of 13 studies showed that the risk of unintentional injuries increases with increasing ambient temperatures. On days with moderate temperatures, the increased risk varied between 0.4% and 5.3% for each 1°C increase in ambient temperature. On extreme temperature days, the risk of injuries decreased. 2 out of 3 studies on occupational accidents found an increase in work-related accidents during high temperatures. For trauma hospital admissions, 6 studies reported an increase during hot weather, whereas 1 study found no association. The evidence for impacts on injuries by subgroups such as children, the elderly and drug users was limited and inconsistent.

**Conclusions:**

The present review describes a broader range of types of unintentional fatal and non-fatal injuries (occupational, trauma hospital admissions, traffic, fire entrapments, poisoning and drug overdose) than has previously been reported. Our review confirms that hot weather can increase the risk of unintentional injuries and accidents in high-income countries. The results are useful for injury prevention strategies.

**Strengths and limitations of this study**

- Robust search strategy including search in three databases.
- Includes all studies published until 07/05/2015.
- Reporting on a broader range of injury outcomes and data sources than in previous studies.
- Evidence reported may not reflect vulnerability in non-high-income settings.

## Introduction

Injuries are a major public health problem in every country of the world. Alone in Europe, intentional (violence and self-harm) and unintentional injuries cause a significant burden of disease each year, accounting for almost 550 000 deaths (6.1% of all deaths) and 10.5% of all disability-adjusted life years in 2011.^1^ In the UK, total injuries accounted for over £23 million pounds in direct health costs to the National Health Service (NHS) in 2013–2014.^2^

Weather factors may have a direct or indirect effect on injuries. A review of the effect of weather on workload at accident and emergency (A&E) departments showed that trauma admissions for children increase in the summer.^3^ It has also been shown that more non-fatal injuries caused by traffic crashes occurred on snowy days compared with dry days.^4^ Falls are also more likely to occur on days with heavy rain compared with light rain, and extreme ambient temperatures can cause direct heat and cold injuries.^5^

The effects of temperature on heat injuries, such as exertional heat stroke, is a well-known occupational health risk.^6^ Human work capacity declines above approximately 26°C due to physiological limitations. High temperatures can also affect cognition, and the risk of mistakes and accidents has been shown to increase during warm weather.^7^ ^8^ In addition, weather may determine a change in behaviour such as participation in outdoor activities.

Since the frequency of high temperature days (HTDs) is expected to increase due to climate change, understanding the impact of high ambient temperature on injuries will become increasingly important and more scientific evidence is needed to develop prevention strategies and improve the planning of healthcare resources. To better inform health policy in the UK, the aim of this review is to summarise and evaluate the epidemiological evidence of the effects of high ambient temperature on unintentional injuries in high-income countries.

## Methods

We conducted a database search to find published studies reporting on the effects of weather on unintentional injuries. We searched three databases (Global Public Health, EMBASE and MEDLINE) by using related keywords and their truncations in titles and abstracts (Weather, season*, climat*, meteorolog*, atmospheric*, humid*, heatwave*, heat-wave*, rainfall*, precipitation, injur*, gash*, slash*, laceration*, contusion*, trauma*, accident*, death*, mortality, hospital admission*, GP*, general practitioner*, ambulance*, emergenc*). Search terms were combined using the appropriate Boolean operator terms. Additionally, we scanned reference lists of identified studies. The search was limited to studies published in the languages English, German and Polish.

Inclusion criteria:

- All studies published until 07/05/2015;
- Studies reporting on fatal and non-fatal unintentional injuries (International Classification of Diseases (ICD)-10 code S00-X59; ICD-9 code 800–999) excluding direct effects of heat, that is, heatstroke (ICD 10 code T67.0–67.7; ICD-9 code 992.0–992.3);
- All age groups for study participants—outcomes by age/sex are reported where possible;
- Studies from high-income countries according to the definition used by the World Bank.^8^

Exclusion criteria:

- Studies reporting only on intentional injuries or heat injuries (ICD-10 code T67.0–67.7; ICD-9 code 992.0–992.3).

We focused on high-income countries since results from low-income and middle-income countries are less likely to be relevant to inform health policy in the UK. Also, we focused on unintentional injuries excluding direct heat injuries as the purpose of this review was to report on accidents rather than on the comparatively rarer occasions of heat illness that have been addressed in previous reviews.^9^ ^10^ ^11^ ^12^

Eligibility assessment was conducted by two reviewers. If the information in titles or abstracts was not sufficient to decide on inclusion or exclusion of the study, the full-text article was retrieved and evaluated. No study was excluded due to disagreement. Information on the selected studies was extracted by one reviewer based on the following items: year of study, study population, exposure and source of exposure data, outcome and source of outcome data, statistical analysis, confounders, study design, sample size, and main findings. Methodological quality assessment was based on epidemiological knowledge and took into account appropriateness of exposure metrics, exposure and outcome data, statistical analysis and reporting of results. Because of the diversity of modelling choices used in the reviewed studies, the results were not quantitatively combined. Only articles reporting the association between summertime ambient temperature and unintentional injuries are presented in this paper.

## Results

**Figure 1:**
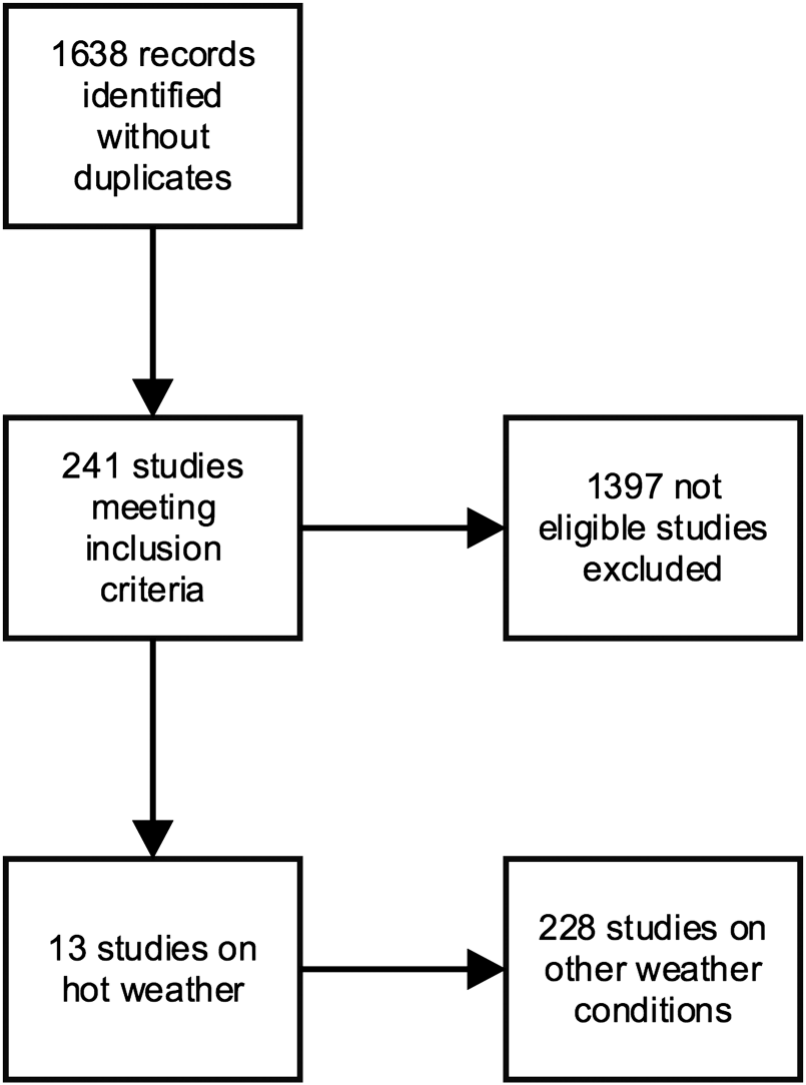
Flow diagram of search.

A total of 1638 papers were identified in the initial search. Based on title or abstract, 241 of these were suitable for full-text review. Thirteen papers met our criteria for this paper (figure 1).

The main findings of the studies are presented in table 1. All studies were retrospective ecological studies. Studies were from Europe (6), North America (5) and Australia (2). The majority used data from hospital settings, but some also used death registration data or wildfire statistics. Study designs were case-series designs or time-series studies which is an approach widely used to investigate the short-term effects of weather on health.^13^ The earliest studies were published in 1998^14^ and 2001.^15^ Eleven studies were performed within the past 10 years. All studies were published in English.

**Table 1:** Reported findings in epidemiological studies regarding high ambient temperature and unintentional injuries

Paper	Study population	Source of exposure data	Exposure metrics	Source of outcome data	Outcome variables	Statistical analysis	Main results
Marzuk *et al*^14^	New York, USA	Hourly temperatures for Central Park	Hot days (maximum hourly temperature of same day or preceding day ≥31.1°C)	Manual review of all medical files at the Office of Chief Medical Examiner for 1990–1995	Unintentional drug overdoses (certified as having died of an unintentional overdose caused by one or more drugs)	Mann-Whitney U test, no account of potential confounders	Mean daily number of unintentional deaths due to drug cocaine overdose was 2.34 on hot days and 1.76 on other days (p<0.001)
Bhattacharyya and Millham^15^	Boston, USA	Daily weather data at Logan International Airport	Maximum daily temperature	Trauma admissions at a level 1 trauma centre (N=9408) from 1992 to 1998	Daily trauma admission volume	Multivariate linear regression controlled for month and season	Pearson r=0.22 (p<0.00 001)
Atherton *et al*^23^	Leicester, UK	Weather data recoded in Newton Linford	Daily maximum and minimum temperature in 5°C intervals	Emergency trauma admissions recorded via the A&E department or via the general practitioner (N=2914) for 1998	Emergency trauma admissions	Poisson regression adjusted for temporal and other weather variables	IRR for maximum temperature: 1.03 (95% CI 0.99 to 1.07; p=0.0001) for total and 1.11 (95% CI 1.03 to 1.19; p=0.0001) for paediatric admissions. IRR for minimum temperature for paediatric admissions was 1.24 (95% CI 1.12 to 1.38; p=0.0001). No information on age group definitions
Morabito *et al*^17^	Florence and Prato, Italy	Hourly weather data from meteorological station	Bio-meteorological index (combined effects of air temperature, relative humidity, wind velocity)	Inpatient discharge data for June, July, September (N=835) for 1998–2003	Work related accidents	Non-parametric (Mann-Whitney U, Kruskal-Wallis H) excluded weekends and August	Variation of accidents over foue temperature quartiles was significant only in June (p=0.008) with the highest mean daily hospital admissions for the 2nd temperature quartile (26.8–29.5°C)
Rising *et al*^20^	Louisville, Kentucky, USA	Hourly temperature data from weather station 4 miles from hospital	10-degree difference in maximum temperature over the preceding 24 h	Registry data of the Trauma Institute of the University Hospital (N=8269) for 1996–2002	Trauma admissions	Poisson or negative binomial regression controlled for trend and annual, weekly and daily variations	5.25% increase in hourly incidents (95% CI 2.5 to 8.09; p<0.001). No information on age group definitions
Rey *et al*^25^	France	Daily minimum and maximum temperature from one weather station per department	‘Heatwave’ period of at least 3 days when the maximum and minimum temperatures were greater than their 95th centile: 30.0°C and 17.3°C	Mortality data from the Inserm (French National Institute for Medical Research) national database for 1971–2003	Injuries and poisoning as an underlying medical cause of death	Age and gender standardised excess mortality estimation	Excess mortality due to injury or poisoning varied across heat waves by 3–14% and was the main cause of death for the <35 years age group except in 2001 and 2003
Nitschke *et al*^18^	Adelaide, Australia	Daily maximum temperatures from Bureau of Meteorology Kent Town (representative for Adelaide)	Heat wave (period in which the daily maximum temperature was 35°C for 3 or more consecutive days, consistent with hotter than usual weather over an extended period)	Daily incidence data of ambulance callouts related to work, road, sport, falls or blunt traumas from the South Australian Ambulance Service for 1993–2006	Ambulance callouts related to work, road, sport, falls or blunt traumas	Conditional fixed-effects Poisson regression adjusted for long-term trend and seasonality (seasonality was controlled for by excluding autumn and winter)	IRR for children aged 5–14 years: sports-related injuries (0.64; 95% CI 0.49 to 0.82), falls (0.60; 95% CI 0.43 to 0.84) and blunt trauma (0.79; 95% CI 0.63 to 1.00) IRR for people aged 75+: motor vehicle-related accidents 0.67 (95% CI 0.47 to 0.97)
Stomp *et al*^19^	Groningen, The Nether-lands	Weather data recorded 10 km from hospital by a meteorological station	Mean daily temperature	Data from the emergency department of the University Medical Centre (N=354 150) for 1970–2005	Daily trauma visit incidence	Poisson regression spline fitting analysis adjusted year, month, weekday and public holidays	IRR 1.15 for each 10°C increase above a threshold 6°C (p<0.05)
Parsons *et al*^24^	England, UK	Temperature data recorded at nearest meteorological station of each centre	Daily maximum temperature	Data reporting injured patients admitted to hospital for 1996–2006 from 21 hospitals (N=59 167)	Daily admission rate relative to the total yearly admission count for each hospital and year	Negative binomial regression models adjusted for effects of year, day of week, week number, public and school holidays	Adults: 1.8% more trauma admissions for each rise of 5°C; paediatric group: 10% more trauma admissions for each rise of 5°C. No age group definitions. No CIs provided
Bohnert *et al*^26^	New York, USA	Temperature data for Central Park	Weekly ambient temperature	Accidental drug overdose deaths in New York City obtained from the Office of the Chief Medical Examiner for 1990–2006	Weekly count of fatal accidental drug overdose	Non-parametric methods with a generalised additive model controlled for year and average length of daylight hours	Non-linear relationship of average weekly ambient temperature with accidental overdose fatality due in whole or in part to cocaine (p<0.05) for a threshold of 24°C, above which overdose deaths increased
Khalaj *et al*^22^	Sydney East; Sydney West; Gosford-Wyong; Newcastle, Illawarra, Australia	Daily minimum and maximum temperature for each monitoring station in the five regions	Extreme heat defined for each region as: Days when temperature exceeded 99th centile of its distribution Same as (A) but for lag=1 Days where the 3-day moving average exceeded its 99th centile	Daily hospital admissions for each of the five regions routinely assembled by NSW Health (N=1 497 655) for 1998–2006	Daily injury emergency hospital admissions	Logistic regression controlled for air pollution (nitrogen dioxide, particulate matter, and ozone) and season	No significant association found Relative odds of emergency hospital admission during extreme heat events for being admitted due to injury compared with being admitted due to another cause: 1.00 (95% CI 0.97 to 1.04) for lag=0, 1.00 (95% CI 0.97 to 1.03) for lag=1, 1.01 (95% CI 0.96 to 1.05) for 3-day moving average
Li *et al*^21^	Milwaukee, USA	Daily weather data from one station in Milwaukee	Mean daily temperature 0–2 days preceding admission	Hospital admissions data provided by all acute care facilities in Milwaukee	Hospital admissions due to accidents and injuries	Time-series analysis controlled for air pollution, relative humidity, day of week, holidays, year and seasonal cycles	1.02% (95% CI 1.00 to 1.05) increase in admissions per 1°C increase for a temperature threshold of 27.2°C (p=0.048)
Cardil *et al*^16^	Spain	Temperature data but no information given on source	HDT (mean air temperature ≥20°C at 850hPa)	General Statistics on wildland fires and wildfire official yearbooks (N=241) from 1980 to 2010	Fire-fighter death	Descriptive proportions, no adjustment for confounding	60% of 241 fire deaths on HTD (5–15% of the total fire season days from June to September)

Studies are ordered by publication date.

A&E, accident and emergency; HDT, high temperature day; IRR, incidence rate ratio; NSW, New South Wales.

### Work-related accidents (three studies)

Three studies reported the effect of hot weather on occupational injuries. Two of these studies found an increase in work-related accidents during increased temperatures, but the other reported no association.

Cardil and Molina^16^ found that 69 (60% of the total) fire-fighter deaths in a wild land fire entrapment occurred on HTDs (see table 1 for definitions) although HTDs represented only a small proportion (5–15%) of the total days in the fire season (June to September) in Spain. An entrapment was defined as either death by hot gases or when a wild land fire reaches the victim. Additionally, fires with a larger number of fatalities occurred under these conditions. Moreover, 60% of terrestrial casualties occurred on HTDs. Terrestrial causality was defined as a person stepping on a power line, casualties while driving a vehicle or falling down with injuries. A possible explanation of the authors’ for these findings was that extreme weather conditions influence fire behaviour and, therefore, increase the probability of entrapment. In addition, these conditions could affect fire fighters due to dehydration, stress and worse working conditions.

An Italian occupational study reported strong evidence (p=0.008) for a variation of work-related accidents over four temperature quartiles in June.^17^ A maximum of 2.1 daily work-related accidents were observed during the third temperature quartile (25.9–28.4°C) when considering the effect of temperature on the day of accident. Their data showed only weak evidence for a difference in the distribution of work-related accidents for the months of July and September or for a delayed effect of temperature by at least 1 day. The authors concluded that hot weather during early summer days may be less well tolerated compared with later summer days due to lack of physiological adaptation to high temperatures. However, another study from Australia found no association, but this was based on relatively higher temperatures (35°C for 3 or more consecutive days), during which people may have adopted preventative measures.^18^

### Hospital admissions for injuries (seven studies)

Five papers reported an increase in trauma hospital admissions during increased temperatures, whereas two papers did not find an association. Three papers analysed paediatric admissions but reported inconsistent results. Three different temperature metrics were assessed. Two papers used mean daily temperature, six papers used daily maximum temperature and two papers used minimum daily temperature.

A Dutch study observed an increase of 15% in trauma patients for each 10°C increase in mean daily temperature above a threshold of 6°C.^19^ For the same difference in maximum temperature over the preceding 24 h, Rising *et al*^20^ found a 5.25% increase in trauma admissions (95% CI 2.50 to 8.09; p<0.001).

In contrast, Bhattacharyya *et al* found only a low correlation with daily maximum temperature (Pearson R=0.22, p<0.00 001).^14^ The study did not report any risk estimate which might still be high despite the low correlation. The departure from the normal (historical average) temperature for the date regardless of season was also slightly correlated with increased trauma admissions (R=0.0923, p<0.0001) implying that hotter than average days add only little to more trauma admissions. The estimated change in risk of 3.61% for a departure from normal of 10F (5.6°C) was not statistically significant. Another US study also reported a small association between high daily temperature and hospital admissions for accidental causes in Milwaukee, Wisconsin.^21^ However, they neither estimated the effect for the index day and each of the two lags separately nor provided any possible mechanism to explain their approach of averaging the temperature over 3 days.

A study from Australia did not find an association between emergency hospital admissions due to injury during extreme heat events (34–38°C daily maximum temperature).^22^

For the UK, Atherton *et al*^23^ found a non-significant association between adult trauma admission and an increase in maximum temperature: 3% (95% CI −1% to 7%; p=0.0001) per 5°C rise. However, they analysed only a short time period (1 year) and data from only one hospital.

A larger and recent UK study reported a lower risk for trauma hospital admissions of 1.8% for the same increase of 5°C in England.^24^ However, they did not report any quantification of the uncertainty or strength of evidence of their estimates.

Both UK studies reported an increased risk for children. For an increase of 5°C minimum temperature Atherton *et al*^23^ reported a rise of 24% (95% CI 12% to 38%; p=0.0001). For an increase of 5°C maximum daily temperature they found a smaller risk of 11% (95% CI 3% to 19%; p=0.0001). Parsons *et al*^24^ estimated a similar risk (10%) for an increase in daily maximum temperature. However, neither UK study reported how age groups were defined. In contrast, the Australian study by Nitschke *et al*^18^ reported decreases during heat waves (defined as periods in which the daily maximum temperature was 35°C for 3 or more consecutive days, consistent with hotter than usual weather over an extended period) in falls (40%; 95% CI 16% to 57%) and blunt trauma (21%; 95% CI 0% to 37%) in children aged 5–14 years.

### Mortality due to injuries or poisoning (one study)

During major heat waves in France, an excess mortality due to injuries and poisoning was observed.^25^ Rey *et al*^26^ reported the estimated proportion of excess mortality due to injuries and poisoning (excluding heat stroke) during a major heat wave as 3–14%. For the 2003 heat wave in particular, they estimated 3% out of 13 734 overall excess mortality. For males under 35 years, except in 2001 and 2003, the majority of the excess mortality was due to injury and poisoning.

### Accidental drug overdoses (two studies)

Two studies investigated the effect of temperature on unintentional drug overdose deaths in New York City (NYC). Marzuk *et al*^14^ investigated the effect of maximum daily temperature and Bohnert *et al*^26^ used average weekly ambient temperature in Central Park. The study population in the Marzuk *et al*^14^ study consisted of all fatal unintentional cocaine overdoses (n=2008) and four comparison groups that included fatal unintentional opiate overdoses (n=793), all other fatal unintentional overdoses (n=85), and a subset of homicides (n=4638) and fatalities from motor vehicle crashes of people aged 15–54 (n=815). Homicides was chosen as the comparison group because they have similar demographics to drug deaths in NYC, and motor vehicle crashes of people aged 15–54 were chosen as a comparison group because of the similar age range. They estimated that the mean daily number of unintentional deaths due to drug cocaine overdose based on maximum daily temperature in NYC was 2.34 on hot days and 1.76 on other days (p value for difference <0.001). There was no evidence for a difference in mean daily deaths due to opiate overdose (p=0.26) or due to overdoses caused by drugs other than cocaine or opiates (p=0.69) on hot days compared with other days. Their study did not find any statistically significant differences by age, race, gender and location between participants who had died on hot days versus another days. However, the sample size may have been too small to detect any differences.

One explanation provided by the authors was that increased temperature places demands on the cardiovascular system to increase output and decrease vascular resistance. The immediate effects of cocaine use include increases in arterial pressure, heart rate and cardiac output. Thus, cocaine use during hot weather may further tax cardiovascular capacity and increase the risk of mortality. Also, diuretics such as mannitol that can be used as cutting agents to dilute cocaine powder could lead to severe dehydration.^27^

The authors rule out that cocaine users take more cocaine on hot days as an alternative explanation because among homicides, which have similar demographics as cocaine overdose cases, cocaine was detected in identical proportions among those dying on hot days versus other days. Also, the proportions of cocaine overdose deaths with a toxicology test result positive for at least one other drug that can reduce heat regulation on hot days versus other days were similar, and rates of screening for drugs are not influenced by weather in NYC.

Bohnert *et al*^26^ analysed a recent period and reported a non-linear relationship of average weekly ambient temperature with accidental overdose deaths due in whole or in part to cocaine (p<0.05). In contrast to Marzuk *et al*,^14^ they estimated a lower threshold of 24°C above which the count of overdose fatality increased. Overdoses where cocaine was present but not cause of death, overdoses due to opiates without cocaine and accidental overdoses due to drugs other than cocaine or opiates did not show an association with ambient temperature. Similarly, weekly deaths due to motor vehicle accidents, where the victim had used cocaine, did not show an association with ambient temperature.

However, the authors of both studies had no or limited data on characteristics of the drug user and drug regime, such as dose of drugs taken before death, frequency of use in the time prior to death and level of tolerance, which may affect the risk of dying from an overdose.

### Hot weather effects on road traffic accidents and sport injuries (one study)

The Nitschke *et al*^18^ study reported decreases during heat waves in Australia in sports-related injuries of 36% (95% CI 18% to 51%) in children aged 5–14 years, and also for motor vehicle-related accidents of 33% (95% CI 3% to 53%) among people aged 75 years and older.^18^ They did not find an association with heat waves in other age groups.

### Methodological approaches of reviewed studies

Injuries are likely to be dominated by time-varying factors. In order to effectively separate them out from the short-term associations between ambient temperature and injuries, it is important to control for key potential time-varying confounders which was dealt differently across studies. Two studies did not adjust for any confounders at all.^14^ ^16^ One study adjusted for weekends,^17^ two studies for month,^15^ ^23^ three studies for public and school holidays,^21^ ^23^ ^24^ five studies for day of week^19^ ^20^ ^21^ ^23^ ^24^ and two studies for weekly variations.^20^ ^24^

Nine studies controlled for major potential temporal confounders such as long-term trend and season but varied in their adjustment approach. Two studies controlled for season by excluding specific months,^17^ ^18^ three studies included season as a factor in the model^15^ ^22^ ^23^ and three studies adjusted for year.^18^ ^20^ ^26^ Only two studies controlled for season and long-term trend using a spline function^19^ ^21^ which is more a flexible method and better able to capture underlying seasonal patterns.

Other potential confounders controlled for were other weather factors,^21^ ^23^ air pollution^21^ ^22^ and daylight.^26^ However, a recent paper suggested that controlling for air pollution in studies investigating temperature health relationships may be problematic.^28^ It was not clear whether controlling for air pollution was necessary in this context. The possibility of delayed effects were assessed by one study for the previous day.^17^

Three of the six time-series studies modelled the temperature injury relationship as a non-linear function^19^ ^21^ ^26^ which based on related studies is likely to be a more appropriate method to estimate the temperature effect than modelling temperature linearly across the whole temperature range, which was done in four studies,^15^ ^18^ ^20^ ^23^ or using descriptive analysis methods such as estimating the proportions of injuries occurring on HTDs, or non-parametric comparisons between hot days and other days^14^ or across different temperature quartiles.^17^ One study estimated excess mortality due to injuries during heat waves,^25^ and Khalaj *et al*^22^ compared the odds of being admitted to a trauma hospital unit during extreme heat events to being admitted due to causes other than injuries. Five studies did not report how many events were analysed^14^ ^18^ ^21^ ^25^ ^26^ and one study did not specify their study period.^21^ Thus, three of the reviewed studies that reported an increase of trauma hospital admissions^19^ ^21^ or accidental drug overdoses^26^ with increasing ambient temperatures were of good quality based on the methods used.

## Discussion

In sum, 11 out of 13 studies showed an increase in unintentional injuries in relation to high temperatures. Temperature thresholds for risks differed depending on the injury outcome and region considered. Seven studies reported evidence for a temperature threshold, ranging from 6°C in the Netherlands for trauma hospital admissions to 31°C in New York, USA, for unintentional drug overdose deaths.

The evidence of effects by age, particularly on vulnerable subgroups such as children and the elderly, is very limited. For children, the evidence was inconsistent, with an increase in injury hospital admissions of 10–24% for each 5°C temperature increase in the UK and a decrease of 21–40% in sport injuries, falls and blunt trauma during heat waves in Australia. Only one study analysed the effect on the elderly, reporting a decrease of 33% in traffic accidents during heat waves in Australia.

For cocaine users as a vulnerable group, two studies from New York, USA, found that unintentional drug overdose deaths increased steadily for temperatures warmer than 24–31°C because high temperatures place additional demands on cardiovascular capacity. In contrast to heat effects on non-communicable disease admissions and mortality, there is a lack of evidence of a delayed effect of increasing temperature on injuries. Only one study analysed lagged effects, reporting weak evidence of an effect of temperature on the previous day.

There are several possible explanations of the observed relationships between increasing temperature and accidents. Changes in behaviour are probably the most common cause, for example, more children playing outside, different driving behaviour and changes in occupational risks (eg, wildfire risks). An alternative explanation is that there is a reduction in skill and power performances during hot weather, leading to tiredness, impaired effectiveness and predisposing individuals to accidents.^29^ ^30^

An analysis of road accidents in Northumberland, UK, showed that most accidents occurred on days with fine weather, although no definition of ‘fine weather’ was provided. Assuming ‘fine weather’ to mean pleasantly warm days, this is in line with findings of our review, with increased trauma admissions on days with moderate summer temperatures, possibly because people are encouraged to go outside. Conversely, Nitschke *et al*^18^ reported a decrease of traffic accidents and injuries during more extreme temperatures, which is plausible since most people may remain indoors during extreme heat. Their results showed a negative effect of temperatures above 35°C in Adelaide. At such temperatures, outside behaviour is limited for most people, particularly those behaviours involving physical activity.^18^ Also, better prevention measures may be in place to prevent injuries during heat waves, especially for school sports.

The results of Nitschke *et al*^18^ are in line with the Morabito *et al*^17^ study that showed a decrease in work-related accidents in Italy for temperatures above 28°C. Behaviour change at very high temperatures to reduce health risks by adopting preventive measures could be one explanation, as well as the initiation of health and safety policies regulating work at high temperatures. The findings of this review on occupational injuries are also consistent with a review on occupational heat exposure reporting that excessive heat exposure remains a significant issue for occupational health.^31^ The Khalaj *et al*^22^ study did not report an association between heat waves and injury emergency hospital admissions in Australia. The Adelaide study by Nitschke *et al*^18^ reported results by age groups and injury type, whereas Khalaj *et al*^22^ reported only an overall association for all ages. Their results may differ if they stratified by injury types and age groups.

Although the majority of the reviewed studies reported an increase of injuries and accidents during increased temperatures, several methodological limitations preclude definite statements on the relationship between injuries or accidents and heat. All reviewed studies used routinely collected data to measure the outcome or databases set up to record occupational health outcomes. To be recorded, an individual must had been admitted to a hospital or trauma centre or had to be recorded in any other database such as the General Statistics on Wildfires. Since not all trauma events are recorded by routine data collection, for instance because they might not have been severe enough for admission or to trigger a visit to an A&E department, under-reporting may underestimate the magnitudes of the temperature effects on injuries and accidents—however, it is likely that all serious injuries (eg, broken bones) would reach hospital, especially in children. No study had information on the exact timing when the injury or accident occurred which could further dilute the true effect. However, since injuries recorded in the analysed data are likely major injuries, it was appropriate to assume that the event happened on the same day since most people with major injuries are unlikely to wait to get admitted to hospital.

All studies used data on temperature from the nearest available weather station. Since temperatures are fairly uniform over a local area and often well correlated between station and location of event, relative changes in temperature are likely to be similar and give a good proxy of the temperature the participants have been exposed to. However, some uncertainty remains to the actual temperatures individuals were exposed to, especially when weather station temperature data are used as a proxy for indoor temperatures. It was not known whether the injuries occurred indoors or outdoors or whether air conditioning was present. The inaccuracy of temperature measurement may have an effect on the estimation of temperature thresholds as well as diluting the estimated effect of temperature on injuries. Individual exposure measures would be more useful—but these were not available in the reviewed studies.

One strength of the present review is that the literature search was run in three different databases. However, as for many reviews, it is very likely that publication bias is present, and thus the reviewed papers presented in this review may not be truly representative of all valid studies. It has been shown that studies reporting weak evidence of an effect are less likely to be published.^32^^–^34 The extent of publication bias is difficult to assess in studies with varied methodology and reporting. However, our aim was not to produce a definitive quantitative estimate of the effect of environmental temperature on injuries, but rather to give an overview of the evidence available. In contrast to a review on health impacts of workplace heat exposure,^10^ the present paper reviewed fewer occupational studies. However, the review by Xiang *et al*^5^ considered studies from low-income and middle-income countries, as well as focusing on heat and occupational health in general and therefore reports on a broader occupational health outcome range. Our review reports on a broad range of injury types (work-related, sport, recreational and traffic accidents) including a wide variety of data sources (inpatient discharges, emergency trauma hospital admissions, ambulance callouts, deaths registries and occupational health registries). Previous reviews of the effects of weather on injuries excluded papers analysing data from death registries or health outcomes such as unintentional drug overdoses and poisoning.

In conclusion, the reviewed studies suggest that increasing ambient temperatures affect admissions to healthcare facilities due to injuries and accidents despite the differences in study methods, study periods and populations. Most workplace and education settings will have legislation about behaviour on days with high ambient temperature and well-established injury prevention strategies in place. However, prevention strategies for other setting such as the Heat Wave Plan for England that currently does not address unintentional injuries^35^ and could be informed by these findings. Health policies, in particular those addressing children, need to be designed carefully to avoid reducing the positive effect on health of increased physical activity outdoors but at the same time to advise on minimising the risk of accidents. To better inform health policy in the UK, more research is needed to estimate region-specific thresholds and risks, with more robust characterisation of risk among the elderly.
